# Metabolic profiling of femoral muscle from rats at different periods of time after death

**DOI:** 10.1371/journal.pone.0203920

**Published:** 2018-09-14

**Authors:** Tieshuai Du, Zebin Lin, Yaling Xie, Xing Ye, Chunyan Tu, Kaidi Jin, Jianhui Xie, Yiwen Shen

**Affiliations:** Department of Forensic Medicine, School of Basic Medical Sciences, Fudan University, Shanghai, China; Oswaldo Cruz Foundation, BRAZIL

## Abstract

Clarification of postmortem metabolite changes can help characterize the process of biological degradation and facilitate investigations of forensic casework, especially in the estimation of postmortem interval (PMI). Metabolomics can provide information on the molecular profiles of tissues, which can aid in investigating postmortem metabolite changes. In this study, liquid chromatography-mass spectrometric (LC-MS) analysis was performed to identify the metabolic profiles of rat femoral muscle at ten periods of time after death within 168 h. The results obtained by reversed-phase liquid chromatography (RPLC)- and hydrophilic interaction liquid chromatography (HILIC)- electrospray ionization (ESI±) have revealed more than 16,000 features from all four datasets. Furthermore, 915 of these features were identified using an in-house database. Principal component analysis (PCA) demonstrated the time-specific features of molecular profiling at each period of time after death. Moreover, results from partial least squares projection to latent structures-discriminant analysis (PLS-DA) disclosed a strong association of metabolic alterations of at least 59 metabolites with the time since death, especially within 48 h after death, which expounds these metabolites as potential indicators in PMI estimation. Altogether, our results illustrate the potentiality of metabolic profiling in the evaluation of PMI and provide candidate metabolite markers with strong correlation with time since death for forensic purpose.

## Introduction

A series of complex physical and chemical changes occur in the organism after death. Characterization of these postmortem alterations is critical for the reliable interpretation of macroscopic and microscopic pathological observation at autopsy [1]. Tomita et al. applied electron microscopy to the observation of postmortem ultrastructural changes in rat tissues, in which corruption with sequential changes was observed in organs comprising pancreas, kidney, liver, heart, and skeletal muscle [2]. Chagnot et al. found that autofluorescence characteristics could be affected by muscle cells undergoing biochemical and physicochemical changes [3]. However, both autolysis and corruption raise difficulties in the estimation of postmortem interval (PMI) based on morphological characteristics of organisms. In fact, the molecular content of mortal remains alters continuously over the time after death. In addition to autolysis, the decomposition of tissues could be initiated by enzymes from postmortem bacteria, which engenders proteins, peptides, amino acids, ammonia, carbon dioxide, amines, hydrogen sulfide, phenol, indole, and methyl indole. An increasing attention has been paid to the elucidation of metabolic profiling in human remains in order to facilitate the investigation of forensic casework [4, 5].

Information at molecular level, from the agonal stage to the supravital and postmortem stage, can be furnished by metabolomics study, as the outcome of cell metabolism and degradation could be profiled at specific periods of time during the process. Such profiles can provide a reliable interpretation of PMI if illustrative biochemical markers are identified and quantified in a general law with the time since death. Metabolic profiling utilizes high-resolution analytical methods, such as nuclear magnetic resonance (NMR) spectroscopy and mass spectrometry (MS), for the quantitative analysis of hundreds of small molecules (<1000 Da) in biological samples [6]. Several studies have been working on the postmortem metabolic characteristics by taking advantages of ^1^hydrogen-nuclear magnetic resonance (^1^H-NMR) [7–9]. Nonetheless, merely components with high abundance in tissues could be identified by means of ^1^H-NMR due to its limited dynamic range and low sensitivity [10]. In contrast, MS analysis has a relatively higher sensitivity, allowing a more comprehensive portrayal of the content of metabolites.

From the analytical perspective, non-targeted metabolomics investigation undertakes a qualitative analysis of metabolites and provides the maximal information of metabolite contents. Several factors should be taken into account to characterize small molecular metabolites in biological samples, such as categories, the polarity span, and the dynamic concentration range. Currently, chromatography-tandem MS has become a pivotal tool in metabolomics. Postmortem metabolites in blood and muscle tissues of animal models were investigated using gas chromatography-mass spectrometry (GC-MS) [11, 12], showing its potentiality in metabolic analysis due to its high-throughput property and high sensitivity [13]. MS, especially liquid chromatography-mass spectrometry (LC-MS), can be used to analyze a wide range of compounds with high sensitivity and reproducibility [14]. As a rapid metabolic analysis technique, LC-MS is suitable for performing untargeted metabolomics in a wide range of fields [15–19]. LC-MS represents a series of analysis platforms that are based on high-performance liquid chromatography (HPLC). Compared to other chromatography-tandem mass spectrometry modalities, LC-MS have more capability of being adapted to the analysis of volatile compounds and poorly heat-stable metabolites. Generally, the speed of analysis of ultra-performance liquid chromatography (UPLC) using a chromatography column filled with 1.7 μm of ultrafine particles is at least 2-fold higher than that of conventional HPLC, and the sensitivity and degree of separation of UPLC are several-fold higher [20]. Ultra-performance liquid chromatography/time-of-flight mass spectrometry (UPLC-TOF MS) has been widely used in metabolomics. Reversed-phase liquid chromatography (RPLC) is suitable for medium or weakly polar metabolites and hydrophilic interaction liquid chromatography (HILIC) has been developed specifically for highly polar metabolites. Previous reports have shown that RPLC- and HILIC-ESI (±)-Q-TOF MS provide maximum information on lipid metabolism and center carbon cycle metabolism, respectively. Especially, the combination of these two separated modes can provide many more metabolite characteristics compared with a single one [21].

Based on the compelling features of maximum information on lipid metabolism and center carbon cycle metabolism, rat femoral muscle tissues were collected at different periods of time after death, and metabolic profiling was performed using LC-MS modalities including RPLC- and HILIC-ESI (±)-Q-TOF MS to explore the different metabolic patterns of postmortem samples. Postmortem metabolic features in rat muscle tissues were further evaluated by principal component analysis (PCA), while potential relationship between metabolic contents with time after death were revealed by partial least squares projection to latent structures-discriminant analysis (PLS-DA).

## Materials and methods

### Ethics statement

All procedures involving animals were performed according to the Guide for the Care and Use of Laboratory Animals and the local institutional ethical guidelines for animal experiments. The protocols were reviewed and approved by the Animal Welfare Committee of the Shanghai Medical College of Fudan University (No. 20140226–021).

### Reagents and animals

Ammonium acetate (NH_4_AC) and ammonium fluoride (NH_4_F) were purchased from Sigma-Aldrich. Formic acid (FA) was purchased from Fluka, and acetonitrile was purchased from Merck.

Sixty male Sprague–Dawley (SD) rats (body weight 213.35±8.72 g) were purchased from the Experimental Animal Center of Fudan University and supplied with water and food ad libitum for three days. The rats were randomly divided into ten groups with six rats each. All animals were euthanized by cervical dislocation and then kept at 25 ± 2°C in a controlled environment chamber. Ten time periods after death were defined: 3, 6, 12, 24, 48, 72, 96, 120, 144, and 168 h. Rats from each group at the indicated time after death were dissected, and skeletal muscle (femoral muscle) was obtained within 30 min at 25 ± 2°C and a humidity of 55 ± 5%. Samples were collected in tubes and stored at -80°C before metabolite extraction.

### Sample preparation

Approximately 60 mg of homogenized femoral muscle (preserved at -80°C; frozen femoral muscle samples were thawed on ice prior to use) was added to 200 μL of cold water and 800 μL of cold methanol/acetonitrile (1:1, v/v) with three magnetic beads in a tube and maintained on ice. Following a simple vortex for 30 s, the reaction was maintained on ice for approximately 10 min. Next, the sample was ground using a FastPrep-24 instrument (4.0 M/S, 120 s) and centrifuged for 5 min (12,000 rpm, 4°C). Then, 600 μL of the upper layer was filtered through a membrane syringe filter (0.22 μm, Nylon 66) and preserved at -20°C. To monitor the stability and reproducibility of the instrument analysis, 20 μL of each sample was used as a quality control (QC) sample and analyzed together with other samples. The supernatant was dried in a vacuum centrifuge. For LC-MS analysis, the samples were redissolved in 100 μL of acetonitrile/water (1:1, v/v) solvent.

### LC-MS/MS analysis (HILIC/MS)

The analyses were performed using an UHPLC (1290 Infinity LC, Agilent Technologies) coupled to a quadrupole time-of-flight mass spectrometer (AB Sciex Triple TOF 6600).

For HILIC separation, the samples were analyzed using a 2.1-mm × 100-mm ACQUITY UPLC BEH 1.7-μm column (Waters, Ireland). In both ESI-positive and ESI-negative modes, the mobile phase contained A = 25 mM ammonium acetate and 25 mM ammonium hydroxide in water and B = acetonitrile. The gradient comprised 85% B for 1 min, a linear reduction to 65% for 11 min, linear reduction to 40% over 0.1 min, maintenance for 2.9 min, a linear increase to 85% over 0.1 min and a 4.9-min re-equilibration time. The gradients were set at a flow rate of 0.3 mL/min, and the column temperatures were maintained constant at 25°C. A 2-μL aliquot of each sample was injected.

For RPLC separation, a 2.1-mm × 100-mm ACQUITY UPLC HSS T3 1.8-μm column (Waters) was used. In ESI-positive mode, the mobile phase contained A = water with 0.1% FA and B = acetonitrile with 0.1% FA; in ESI-negative mode, the mobile phase contained A = 0.5 mM ammonium fluoride in water and B = acetonitrile. The gradient comprised 1% B for 1.5 min, a linear increase to 99% over 11.5 min and then maintenance for 3.5 min. Subsequently, the gradient was reduced to 1% over 0.1 min, and a 3.4-min re-equilibration time was employed. The gradients were set at a flow rate of 0.3 mL/min, and the column temperatures were maintained constant at 25°C. A 2-μL aliquot of each sample was injected.

After HILIC separation, the ESI source conditions were adjusted as follows: Ion Source Gas1 (Gas1) as 60, Ion Source Gas2 (Gas2) as 60, curtain gas (CUR) as 30, a source temperature of 600°C, and IonSpray Voltage Floating (ISVF) ± 5500 V. For MS-only acquisitions, the instrument was set to acquire data over the *m/z* range 60–1000 Da, and the accumulation time for the TOF MS scan was set at 0.20 s/spectra. For auto MS/MS acquisitions, the instrument was set to acquire more than the *m/z* range of 25–1000 Da, and the accumulation time for the product ion scan was set to 0.05 s/spectra. The product ion scan was acquired using information-dependent acquisition (IDA) with high sensitivity mode selected. The parameters were set as follows: a collision energy (CE) fixed at 35 V ± 15 eV, a declustering potential (DP), 60 V (+) and −60 V (−), an exclusion of isotopes within 4 Da, an exclusion time of 4 s and 6 candidate ions to monitor per cycle.

After RPLC separation, the ESI source conditions were set as follows: Gas1 40, Gas2 as 80, CUR as 30, a source temperature of 650°C, and ISVF ± 5500 V. For MS-only acquisitions, the instrument was set to acquire over the *m/z* range of 60–1000 Da, and the accumulation time for the TOF MS was set to 0.20 s/spectra. For auto MS/MS acquisitions, the instrument was set to acquire over the *m/z* range of 25–1000 Da, and the accumulation time for the product ion scan was set to 0.05 s/spectra. The product ion scan was acquired using IDA with high sensitivity mode selected. The parameters were set as follows: a CE fixed at 35 V ± 15 eV, a DP, 60 V (+) and −60 V (−), the exclusion of isotopes within 4 Da, an exclusion time of 4 s and 6 candidate ions to monitor per cycle.

### Metabolite identification

The raw MS data (wiff.scan files) were converted to mzXML files using ProteoWizard MSConvert before being imported into the open-source XCMS software. For peak identification, the following parameters were used: centWave *m/z* = 25 ppm, peak width = c (10, 60), and prefilter = c (10, 100). For peak grouping, the following parameters were employed: bw = 5, mzwid = 0.025, and minfrac = 0.5. In the extracted ion features, only the variables exhibiting more than 50% of the nonzero measurement values in at least one group were retained. Compound identification of the metabolites was performed by comparing the accuracy of the *m/z* value (<25 ppm) in the MS/MS spectra with an in-house database that was established with the available authenticated standards [22]. In-house database is a metabolite tandem spectral library with about 1500 metabolites, and most of these metabolites are polar compounds.

### Statistical analysis

After the normalization of the total peak intensity, SIMCA-P V13.0 (Umetrics, Sweden) was then introduced for further analysis of the data. PCA, PLS-DA, and orthogonal PLS-DA (OPLS-DA) were undertaken for both positive and negative model construction after log transformation and Pareto scaling. In the PCA, all QC samples were well assembled, confirming the stability of LC-MS. The variable importance in the projection (VIP) value of each variate in the PLS-DA model was calculated so as to indicate the modeling importance of every variate and meanwhile their influence on the response variables [23]. The predictive ability of the developed model was evaluated using a permutation test. To ensure the stability of the probability estimates, 20 permutations were carried out. In SIMCA P+, VIP plots are sorted based on the importance of variables. Variables with VIP of > 1 are usually considered the most important for explaining Y-variables. To obtain metabolites that were highly associated with PMI, the metabolites were screened with a threshold of VIP score >1.5. Metabolites with VIP values of >1.5 were analyzed by Student’s t-test at the univariate level, and those with p-values <0.05 were considered statistically significant [22].

## Results

### Overview of metabolites in rat femoral muscle after death

**Fig 1:**
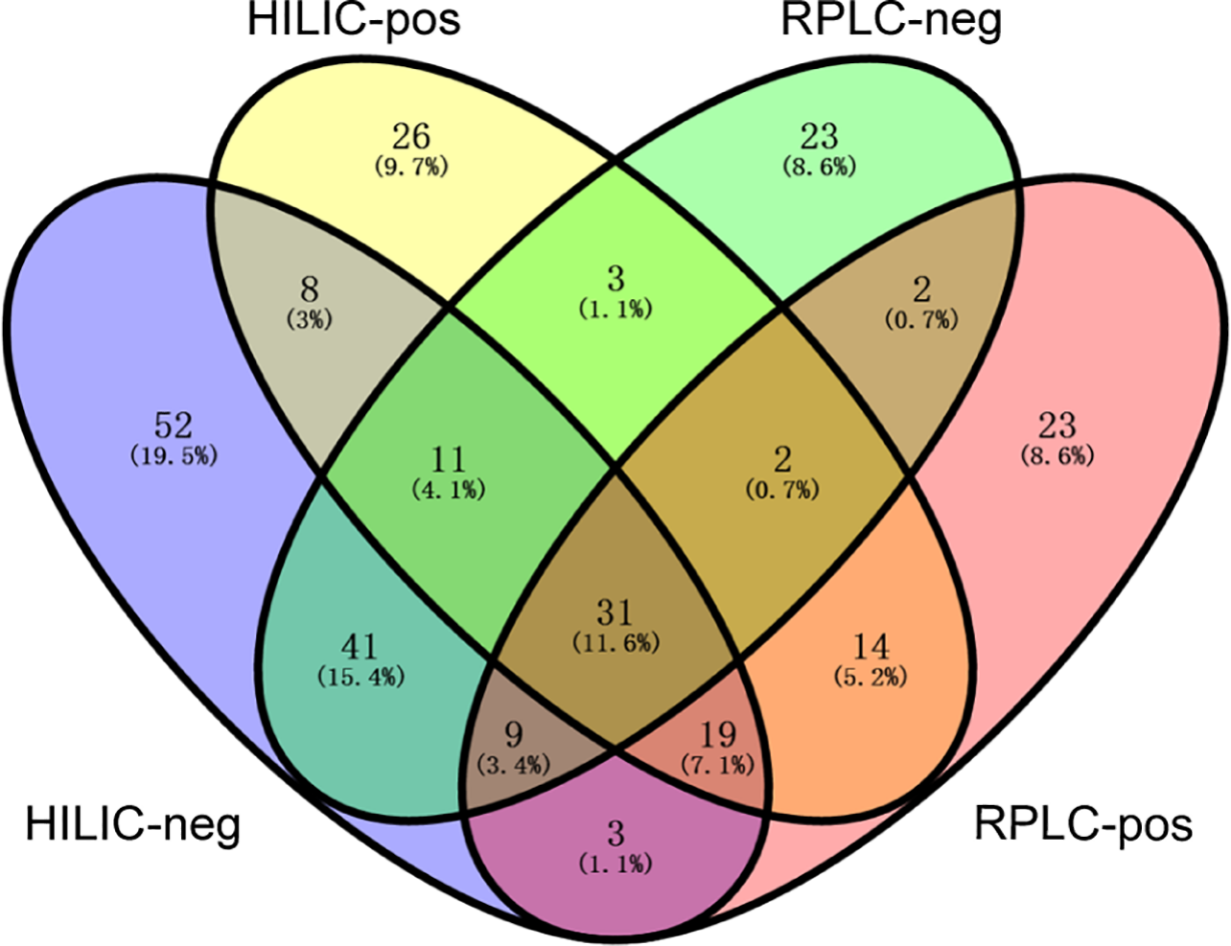
Metabolites from the four datasets identified in this study. HILIC-neg indicates the dataset obtained by hydrophilic interaction liquid chromatography (HILIC)-electrospray ionization (ESI) in negative mode. HILIC-pos indicates the dataset obtained by HILIC-ESI in positive mode. RPLC-neg indicates the dataset obtained by reversed-phase liquid chromatography RPLC-ESI in negative mode. RPLC-pos indicates the dataset obtained by RPLC-ESI in positive mode.

LC-MS including RPLC- and HILIC-ESI (±)-Q-TOF MS was performed using samples from ten periods of time after death, and features from all four datasets were obtained (Table 1). A total of 16,000 features were extracted from all four datasets and approximately 5.5% of all features (915 features) were simultaneously identified using an in-house database (Table 1). Primary metabolites from major metabolic pathways, such as amino acids, nucleotides, and organic acids, were included in these sets. Results of corresponding features characterized in each dataset are presented in a Venn diagram, which suggests that some of these features appeared specifically in a single group yet others in the intersection of groups (Fig 1). Briefly, thirty-one metabolites appeared in all four datasets, while 124 metabolites displayed an exclusive pattern. Furthermore, HILIC-ESI (-)-Q-TOF MS revealed many more features than the other three LC-MS modalities, and most of these features were reproduced by the other three LC-MS modalities (Fig 1).

**Table 1:** Data from RPLC- and HILIC-ESI (±)-Q-TOF MS.

Dataset	Number of Features	Identification Number #	Samples outside the 95% Confidence Interval (PCA analysis)
HILIC-pos	3522	219	3h.4ǂ, 48h.3, 168h.4
HILIC-neg	4823	338	48h.3, 144h.3, 144h.5, 168h.4
RPLC-pos	4771	173	120h.3, 144h.3, 168h.4
RPLC-neg	3468	185	72h.3, 96h.6, 120h.3, 144h.3, 168h.4

#The number of metabolic features identified in the in-house database

ǂThe sample was nominated with both PMI and the intragroup sample number before and after the dot.

**Fig 2:**
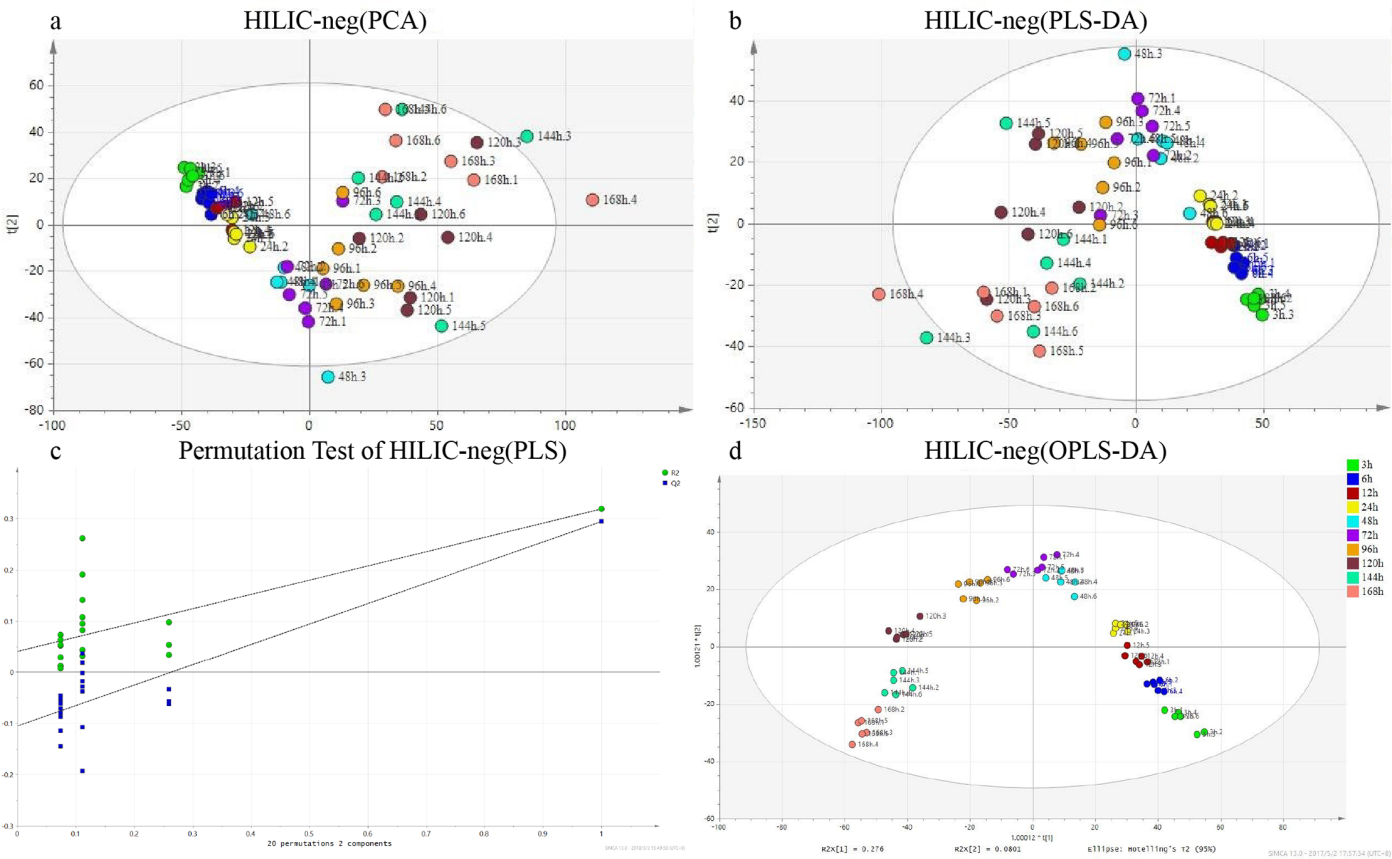
Metabolic analysis of the HILIC-neg dataset. (a) PCA analysis. The ellipse was obtained by Hotelling’s T2 with a 95% confidence interval. (b) PLS-DA analysis. (c) The permutation test plot in PLS-DA. (d) OPLS-DA analysis. Different colors were used to indicate the various periods of time after death. Each sample was nominated with both PMI and the intragroup sample number before and after the dot.

Subsequently, PCA analysis was performed using the dataset “HILIC-neg”. As shown in Fig 2A, minimal diversity was observed in the metabolic profiles of samples obtained within 48 h after death and however a significant separation was observed in samples with an interval of more than 48h after death. In the PCA plot, several samples were observed outside the 95% confidence interval (Fig 1A and Table 1). These results were further verified by the PLS-DA analysis (Fig 2B), which were consistent with those of previous reports [11, 12]. In the permutation test plot, the y-intercept values of the regression line were 0.0413 for R2 and −0.105 for Q2 (Fig 2C). In contrast, the OPLS-DA analysis clustered all samples at each period of time after death (Fig 2D). Similar results were observed in the PCA, PLS-DA, and OPLS-DA analysis using the HILIC-pos, RPLC-pos, and RPLC-neg datasets, respectively (data not shown).

### The relationship between the molecular profile and PMI

**Fig 3:**
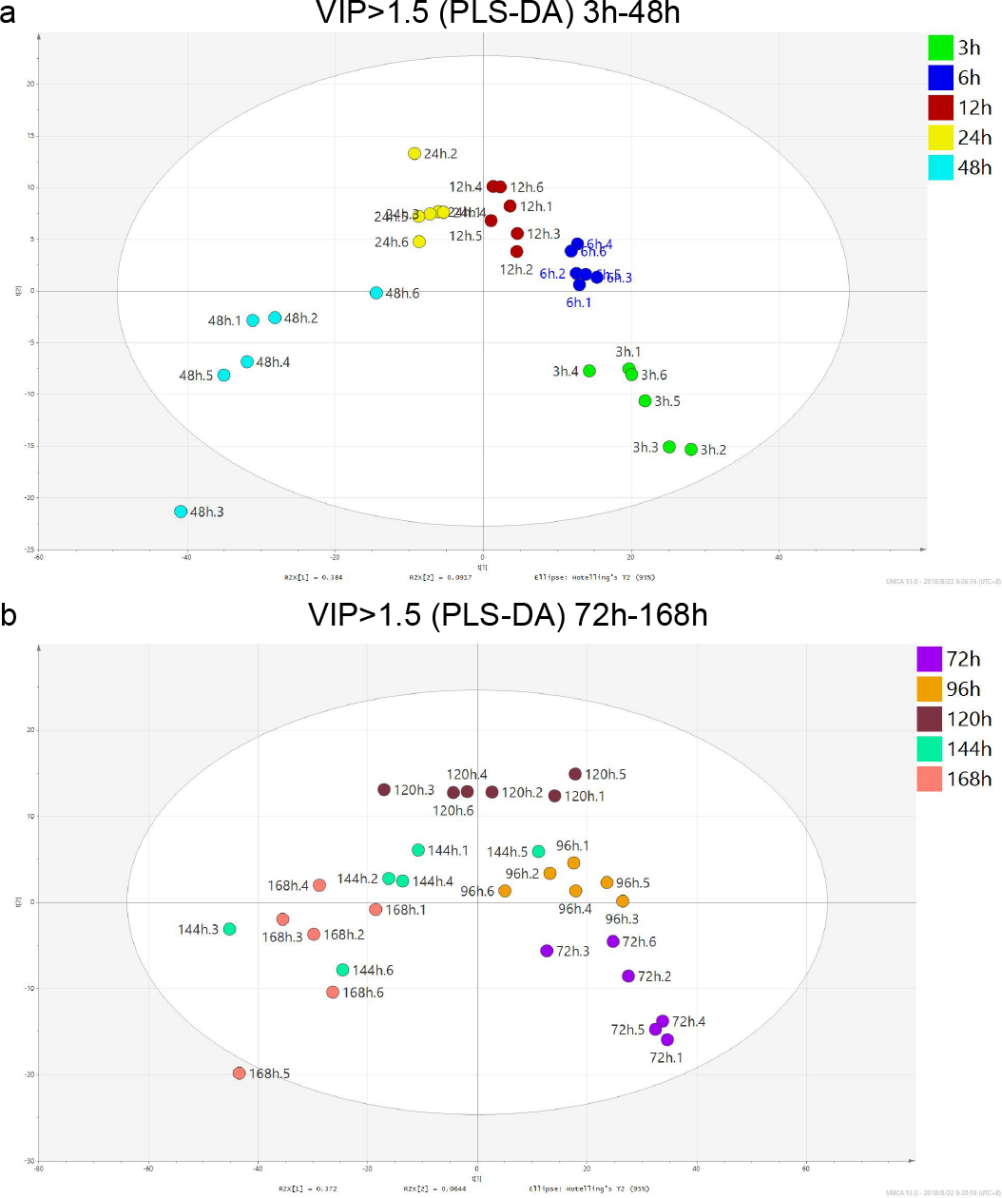
PLS-DA analysis of 1067 features with VIP values of >1.5. (a) PLS-DA analysis of samples within 48 h after death. (b) PLS-DA analysis of samples from 72 h to 168 h after death. Different colors were used to indicate the various periods of time after death. Each sample was nominated with both PMI and the intragroup sample number before and after the dot.

Based on the PLS-DA analysis, the features with VIP values of > 1.5 in each dataset were collected, and a total of 1067 features were obtained after filtering. PLS-DA was further performed with these 1067 features. As shown in Fig 3A, the metabolic profiles of the samples obtained within 48 h after death were clustered. On the contrary, the metabolic profiles of the samples with an interval of more than 48 h after death showed a large diversity (Fig 3B).

**Fig 4:**
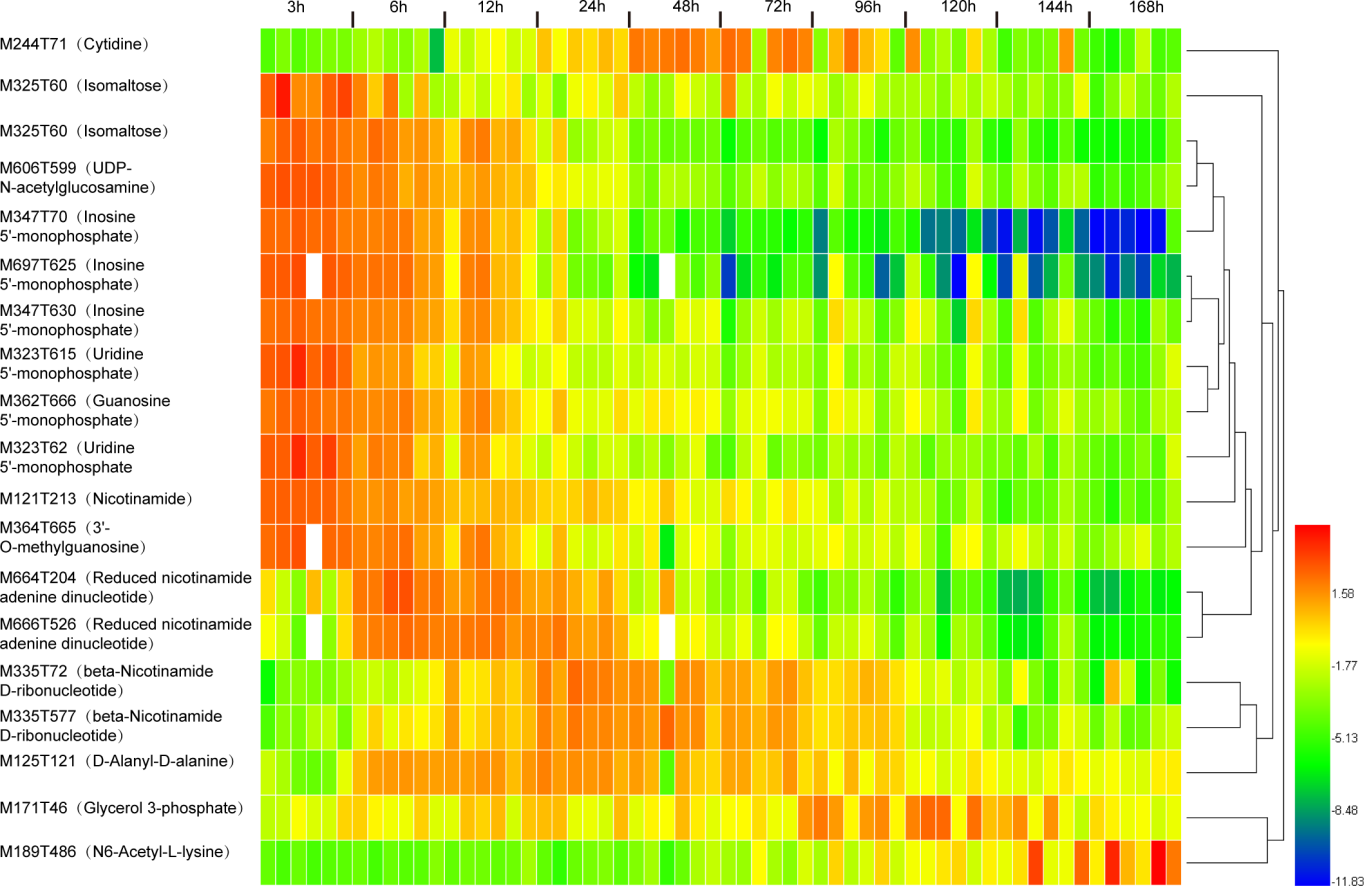
Heat map of the identified metabolites. Substances have different patterns under controlled experimental conditions at specific periods of time after death.

Metabolites from the obtained 1067 features were further filtered by PLS-DA analysis with VIP values of > 1.5. A total of 59 metabolites with VIP values of > 1.5 were obtained after the second PLS-DA analysis and most of them were concurrently embodied in multiple datasets. Among them, 19 metabolites were verified via comparisons to the in-house database (Fig 4 and Table 2) and the other 40 metabolites remained unknown in this study (Table 3). Despite their undefinable nature in this study, a close association was inclined of them with the time after death.

**Table 2:** Characterized metabolites that were highly related to the PMI.

Name	Adduct	Description	*m/z*	rt (min)
M323T615	(M-H)-	Uridine 5'-monophosphate	323.0267	10.25
M606T599	(M-H)-	UDP-N-acetylglucosamine	606.0726	9.98
M347T630	(M-H)-	Inosine 5'-monophosphate	347.0391	10.50
M362T666	(M-H)-	Guanosine 5'-monophosphate	362.0482	11.11
M335T577	M-	Beta-nicotinamide D-ribonucleotide	335.0639	9.61
M364T665	(M-2H+3Na)+	3'-O-methylguanosine	364.0633	11.09
M666T526	(M+H)+	Reduced nicotinamide adenine dinucleotide	666.1303	8.77
M189T486	(M+H)+	N6-acetyl-L-lysine	189.1216	8.11
M125T121	(M+H-2H_2_O)+	D-alanyl-D-alanine	125.0690	2.02
M697T625	(2M+H)+	Inosine 5'-monophosphate	697.1012	10.41
M325T60	(M+H-H_2_O)+	Isomaltose	325.1103	1.01
M244T71	(M+H)+	Cytidine	244.0926	1.18
M349T72_2	(M-2H+3Na)+	2'-O-methylinosine	349.0537	1.19
M323T62	(M-H)-	Uridine 5'-monophosphate	323.0291	1.04
M664T204	(M-H)-	Reduced nicotinamide adenine dinucleotide	664.1191	3.40
M121T213	(M-H)-	Nicotinamide	121.0412	3.55
M347T70	(M-H)-	Inosine 5'-monophosphate	347.0405	1.16
M171T46	(M-H)-	Glycerol 3-phosphate	171.0055	0.77
M335T72	M-	Beta-nicotinamide D-ribonucleotide	335.0657	1.20

**Table 3:** Uncharacterized metabolites that were highly related to the PMI.

Name	*m/z*	rt (min)	Name	*m/z*	rt (min)
M320T95	320.052	1.58	M560T671	560.3543	11.18
M350T73	350.0564	1.21	M334T526	333.5689	8.77
M179T72	179.0472	1.19	M320T575	320.0521	9.59
M337T269	337.1377	4.48	M337T576	337.0786	9.59
M263T340	263.0839	5.67	M664T605	664.1145	10.08
M206T155	206.0476	2.58	M276T235	276.1789	3.92
M332T204_1	331.5559	3.40	M377T44	376.9667	0.74
M377T488	376.9687	8.14	M251T44_1	250.9752	0.74
M744T56	744.3762	0.94	M662T604	662.0988	10.07
M300T239	300.0997	3.98	M543T642	542.9899	10.71
M318T240	318.11	4.00	M219T290	219.0308	4.84
M613T56	613.3053	0.93	M445T641	445.0135	10.68
M341T57	341.1091	0.95	M341T494	341.1067	8.23
M261T346	261.0707	5.77	M601T641	601.1351	10.68
M158T477	158.0615	7.95	M242T205	242.078	3.42
M227T239	227.05	3.99	M665T750	665.2124	12.49
M202T235	202.0546	3.92	M87T69	87.00782	1.16
M267T71	267.1058	1.19	M171T67	171.102	1.11
M609T660	609.3569	11.00	M234T63	234.0764	1.05
M584T640	584.3543	10.67	M215T391	215.0177	6.51

**Fig 5:**
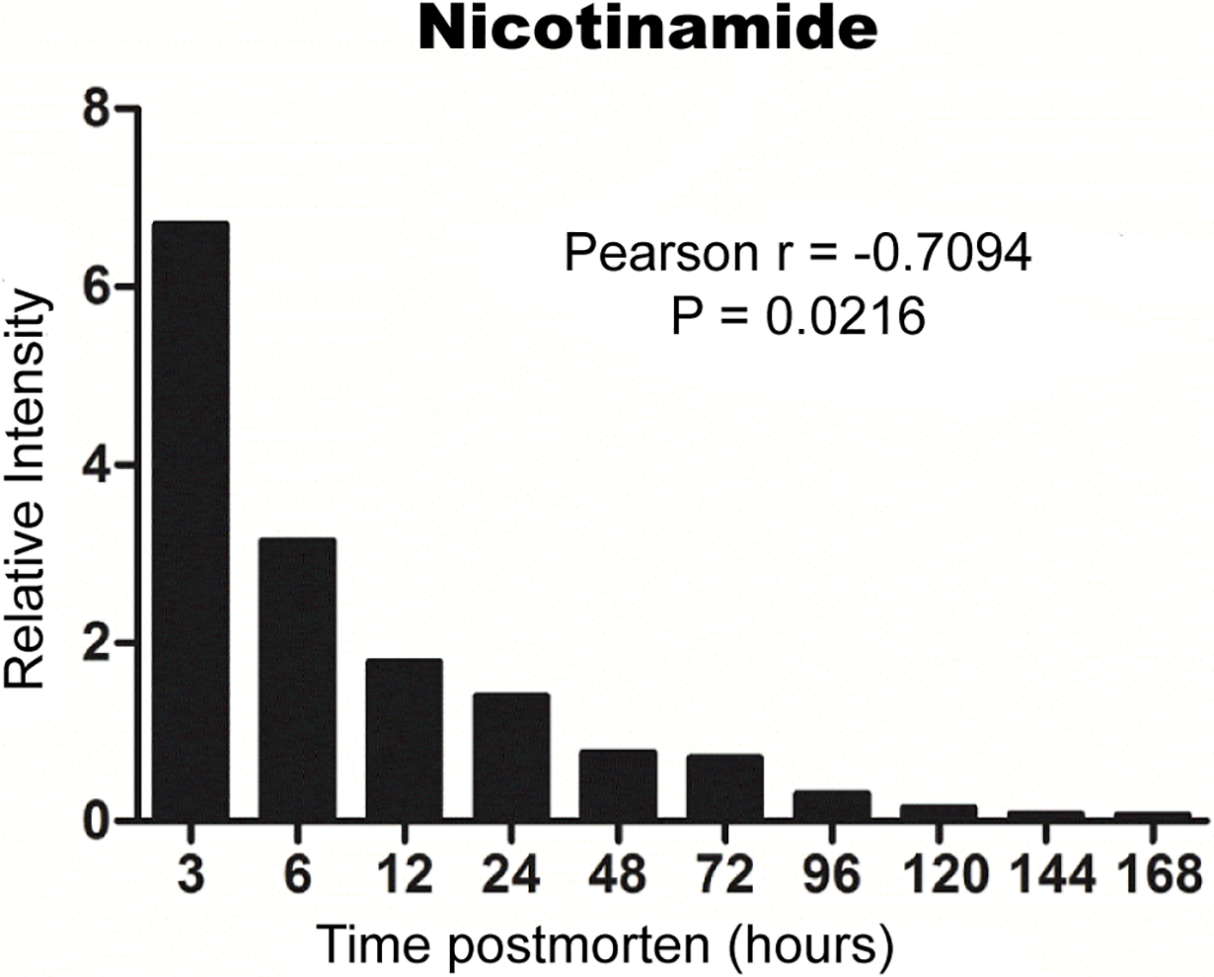
The relative intensity of Nicotinamide at corresponding periods of time after death. An apparent descending tendency could be observed in the whole process within 168 h after death, while the decline became implicit after 48 h.

**Fig 6:**
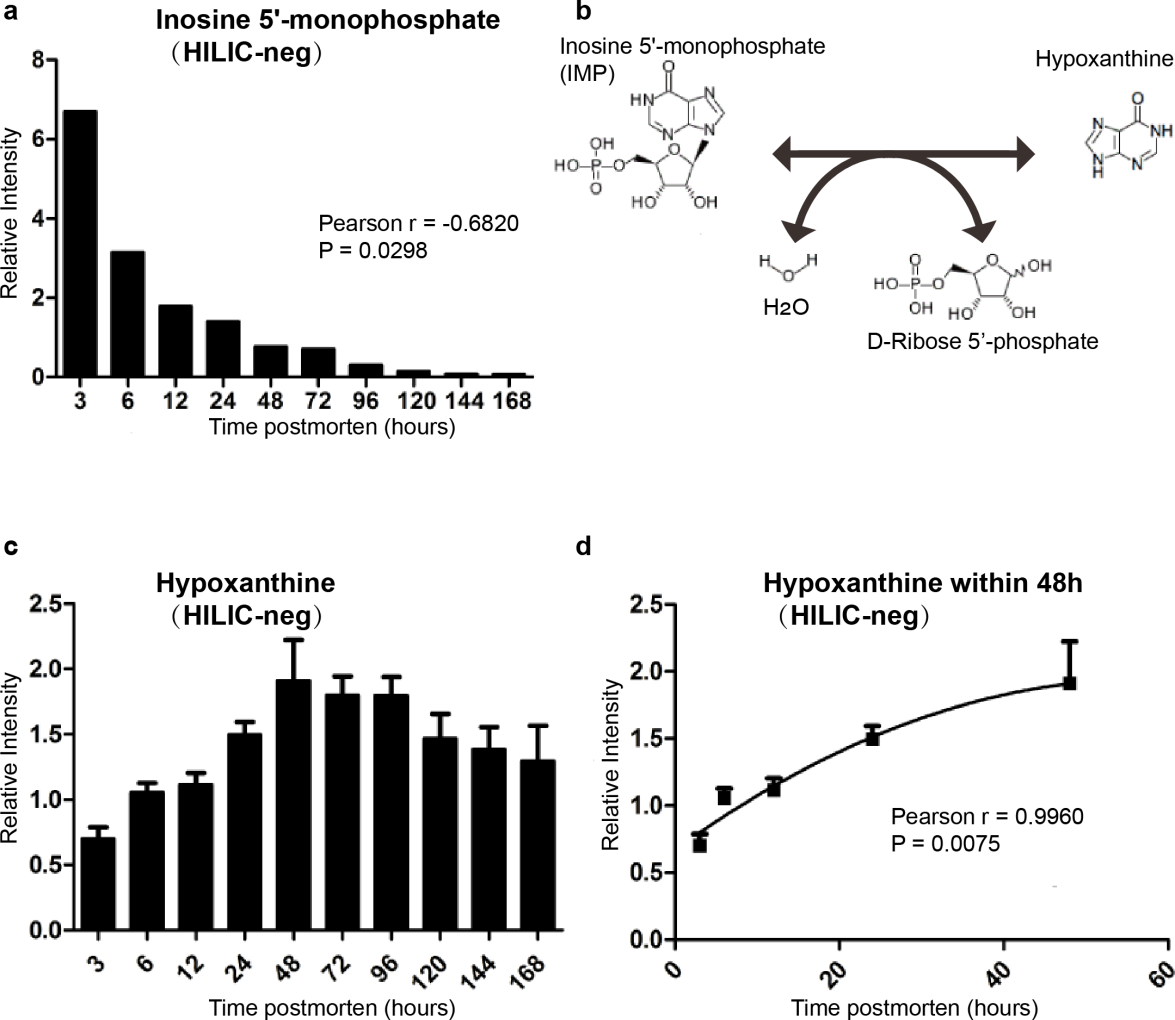
The relative intensity of representative metabolites at corresponding periods of time after death. (a) The relative intensity of inosine 5'-monophosphate (IMP) over time after death. (b) The interconversion of IMP and hypoxanthine. (c) The relative intensity of hypoxanthine over time after death. (d) The relative intensity of hypoxanthine within 48 h after death.

Subsequently, a composition change analysis was carried out and the results showed that different substances exhibited dissimilar trends over PMI (Fig 4). N6-acetyl-L-lysine increased with the time of death, while nicotinamide (Fig 5) and inosine 5'-monophosphate (IMP) (Fig 6) decreased. Beta-nicotinamide, D-ribonucleotide, and nicotinamide adenine dinucleotide (NADH) rose at the beginning and declined afterwards. The trend of each metabolite might be attributed to their different metabolic pathway after death. Generally, the content of all endogenous metabolites still conformed to a downward trend as the increasing time after death.

## Discussion

Metabolic profiling can obtain higher information content compared with morphological observation, which helps in the establishment of the relationship between postmortem changes in metabolites and the time after death. Thousands of metabolites linked to the time since death can be revealed by metabolic approaches and meanwhile information of changes in metabolites after death can also be enriched. RPLC- and HILIC-ESI (±)-Q-TOF MS can complement one another in metabolic profiling and the combination of the two individual modes can provide a full-scale characterization of metabolites [21]. Four datasets provided complementary postmortem metabolic information (Fig 1). Notably, sample No. 3 in the 144-h group and sample No. 5 in the 168-h group appeared out of the 95% confidence interval in the PCA analysis with the four datasets. Similar results were acquired with the PLS-DA analysis in the single-group experiment (Fig 2). It might be in that individual differences could barely be evitable, especially under circumstances that metabolites in the body will undergo sophisticated and substantial changes as the time after death and the decomposition process progresses.

In previous preliminary studies concerning PMI, plasma from SD rats as well as muscle and serum from mice were analyzed by GC-MS to determine metabolic profiles [11, 12]. Those studies focused on the PMI within 48 h and proposed the preliminary inference model [11, 12]. In this study, PCA, PLS-DA, and OPLS-DA were used to discriminate samples from different periods of time after death (Fig 2). Although the metabolites contained herein were not the same as those obtained by the GC-MS studies, the results of multivariate analysis has shown a similar pattern. In addition, conclusion could be drawn that within 48 h, a higher level of distinctiveness between each group and a tighter cluster of samples within each group were evident, which leads to a significant discrimination among different periods of time after death. In contrast, when the interval after death exceeded 48 h, there was some overlap among each group, especially between 144-h and 168-h groups. This might be related to a saturation of changes in the samples, which occurs after 48 h since death. Another possibility could be in that tiny individual differences had been enlarged by the time after death and such differences were not associated with PMI.

In the PCA analysis, most of the samples appearing outside the 95% confidence interval were of groups with an interval of more than 48 h after death (Table 1) and results of the PLS-DA analysis showed a conspicuous deviation. Consequently, the period of 48 h after death can be considered as a critical point of degradation-based analysis. We speculate that the body temperature becomes similar to the ambient temperature after 24–48 h. At this stage, rigor mortis will pass, and algor mortis will eventually result in the equilibration of the body temperature with the ambient temperature. Therefore, molecular changes were not significantly associated with PMI after the first 24–48 h period.

A high correlation is evident among the variables, and different data screening methods obtained similar results. In this study, we adopted the conventional methods of the PCA and PLS-DA analysis with the VIP value for data filtering. A threshold (VIP>1.5) was used to obtain metabolites closely related to PMI in this study. Consequently, fifty-nine metabolites were observed to exhibit significant changes according to the time after death (Tables 2 and 3). These metabolites were highly related to the time since death according to our investigation and could be considered candidate metabolic markers for the evaluation of the PMI. The results of the heat map [24] analysis demonstrated different change patterns of various substances under controlled experimental conditions (Fig 4). The contents of isomaltose, 2'-O-methylinosine, and IMP were initially high and then gradually decreased over time, while reduced NADH and D-alanyl-D-alanine had a low level at the beginning and increased after 6 h since death, followed by a gradual decreased after 48 h. Therefore, postmortem metabolite changes are clearly evident in the femoral muscles of rats.

A PMI estimation using only postmortem morphological changes by forensic pathologists leads to great deviation, particularly for a prolonged postmortem process [25]. Although judicial doctors can also utilize the temperature of the dead body to make judgments [26], the disadvantage is the similarity of the ambient temperature to the body temperature, which makes an accurate determination of PMI (generally within 24 h) quite difficult. While metabolites vary at different periods of time after death, the PLS-DA and OPLS-DA analyses showed that the degree of dispersion of samples with an interval of more than 48 h was greater than that within 48 h. Interestingly, two previous studies have made a similar conclusion that the metabolites in the animals only lasted up to 48 h after death [11, 12]. Therefore, metabolomics can allow a model-based analysis of PMI within 48 h.

Biochemical changes in vitreous humor have been used to estimate the PMI for more than 40 years based on topographical isolation and adequate protection, and the level of potassium ions was previously the only independent variable for regression analysis [27–30]. With advancements in this field, the effects of magnesium and hypoxanthine in vitreous humor have also been considered to be superior to the effect of potassium ions in vitreous humor [31–33]. However, as shown in Table 2, we did not obtain hypoxanthine in PLS-DA analysis. In fact, hypoxanthine was obtained in the raw data and single-compound analysis revealed an initial rising trend followed by a decline after 48 h (Fig 6C). Although the content of hypoxanthine had exhibited differences among different periods of time after death, the trend was not distinct from those of other obtained metabolites. Meanwhile, IMP, an intermediate in the degradation of purines and purine nucleosides and in pathways of purine salvage, was obtained in the HILIC-neg/HILIC-post/RPLC-neg datasets. We observed the same degradation trend of hypoxanthine in the three other datasets and that the change of hypoxanthine was not significant after 48 h (Fig 6D). Therefore, we speculate that hypoxanthine has been filtered out by stringent screening conditions according to the change with the time after death. The main aim of all such studies is to develop a target substance-varying model for predicting the time after death. Even though we identified a more effective substitute factor for the predictive model, a single substitute was not sufficient for predicting the time of death. Therefore, we suggest that a set of multiple parameters could be used to increase the accuracy of PMI evaluation, and LC-MS might be an adequate analysis technique.

The molecular content of mortal remains alters continuously over the time since death and metabolomics has great advantages in postmortem research. High-throughput analysis techniques have been applied in many fields and are believed to be useful in the analysis of postmortem metabolite changes [15–19]. Specifically, LC-MS analysis can obtain 10-fold more data than GC-MS analysis, indicating many more metabolic changes. Although most metabolites have not yet been identified, multivariate statistical analysis can identify features in the LC-MS dataset that exhibit differences in signal intensity among samples, thereby potentially corresponding to the biomarkers [34]. Herein, we present results of LC-MS analysis similar to that of previous reports and propose some metabolites that could be useful markers for estimating the PMI. To our knowledge, this is the first investigation to reveal changes in metabolites after death in a rat model using LC-MS/MS-based metabolic profiling.

## Supporting information

S1 Table HILIC-neg dataset. (CSV)

S2 Table HILIC-pos dataset. (CSV)

S3 Table RPLC-neg dataset. (CSV)

S4 Table RPLC-pos dataset. (CSV)
